# Seeing Life through Positive-Tinted Glasses: Color–Meaning Associations

**DOI:** 10.1371/journal.pone.0104291

**Published:** 2014-08-06

**Authors:** Sandrine Gil, Ludovic Le Bigot

**Affiliations:** University of Poitiers and CNRS (CeRCA, UMR 7295), Poitiers, France; Birkbeck, University of London, United Kingdom

## Abstract

There is a growing body of literature to show that color can convey information, owing to its emotionally meaningful associations. Most research so far has focused on negative hue–meaning associations (e.g., red) with the exception of the positive aspects associated with green. We therefore set out to investigate the positive associations of two colors (i.e., green and pink), using an emotional facial expression recognition task in which colors provided the emotional contextual information for the face processing. In two experiments, green and pink backgrounds enhanced happy face recognition and impaired sad face recognition, compared with a control color (gray). Our findings therefore suggest that because green and pink both convey positive information, they facilitate the processing of emotionally congruent facial expressions (i.e., faces expressing happiness) and interfere with that of incongruent facial expressions (i.e., faces expressing sadness). Data also revealed a positive association for white. Results are discussed within the theoretical framework of emotional cue processing and color meaning.

## Introduction

Colors carry information that goes far beyond esthetics, owing to their emotionally meaningful associations. They can therefore transcend their physical nature and take on a psychological meaning [1]. As such, colors can be regarded as a relevant informational context in the processing of all types of stimuli. The present study was designed to investigate the positive meaning of two colors (i.e., green and pink), and its influence on the everyday mechanism of emotional face processing.

Emotional facial expressions provide critical cues in human interactions, as they convey information both on other people’s states and on the environment (e.g., [2]–[4]). According to many emotion theorists, each discrete emotion is expressed via a specific pattern of facial muscles, and can be accurately understood by humans [5]–[7]. Undoubtedly, the ability to understand these nonverbal communication cues allows humans to engage in harmonious interactions and implement adaptive behaviors. The automaticity perspective on emotion recognition is driven by the basic emotion approach (e.g., [8]–[10]). Psychology researchers have therefore focused mainly on the human ability to understand these nonverbal messages, highlighting extremely efficient processes in adults that seem to be both automatic and effortless (e.g., [11]–[14]).

A relatively recent criticism of these classic studies concerns their nonecological design: previous investigations have mainly focused on emotional faces perceived in isolation, whereas in everyday life, humans see these faces in context. Here, *context* refers to contextual information, in other words, stimuli that are present alongside a target stimulus, and which can modulate (i.e., constrain or facilitate) the processing of that target [15]. Research findings are increasingly showing that emotional contextual information influences affective face processing in different types of context [16], [17]. As such, the influence of external features (i.e., features that are not intrinsically linked to the expresser’s body and face) has been a recent focus of interest, and has so far been tackled in three different ways. The first involves the manipulation of a context described verbally (e.g., [18], [19]). For example, in an fMRI investigation, participants who looked passively at faces expressing surprise (i.e., an ambiguous emotion per se that can be either positively or negatively interpreted) showed a difference in amygdala activation reflecting the negative or positive interpretation of the faces as a function of the valence of a priming sentence [20]. The second involves examining the influence of faces forming the context for the interpretation of a target face. This has revealed a decrease in emotional face recognition performance when a conflicting emotional expression is in its periphery [21], and an increase when a congruent one is present [22]. The third focuses on the influence of emotional scenes forming the background in emotional face recognition tasks [23]–[27]. For instance, ambiguous fearful facial expressions are more efficiently categorized as displaying fear when they are displayed with an emotional scene conveying the same (i.e., negative) emotion, rather than a neutral or positive one [24], thus demonstrating the so-called *congruency effect:* 1/facilitated face recognition when the face and contextual features convey the same emotion, leading participants to be faster and/or more accurate in recognizing the emotional face; or, conversely, 2/impaired recognition performance when the facial and contextual features convey contradictory emotions, creating interference.

Color is one of the most ubiquitous features of the environment. It can be intrinsically linked to the processing of any object that is perceptible to humans, and can thus be assumed to influence information processing. Therefore, it makes sense for researchers to investigate the impact of color on behavior and psychological functioning [28]. Studies in different fields have revealed how color can influence our perception, affect and cognition, demonstrating, for instance, the existence of perceptual confusion between color and odor (e.g., [29], [30]), or the impact of color on internet use (e.g., [31], [32]). In the present study, we reasoned that color (or more precisely hue, when controlling for lightness and saturation) has an impact on psychological functioning because it carries information arising from its emotionally meaningful associations. While research on the link between color and psychological functioning is nothing new (e.g., [33]), Elliot and colleagues recently developed a theoretical model of this link [34], [35]. This model hypothesizes that color conveys a specific message that can be explained either phylogenetically (i.e., colors convey biologically-based messages), or ontogenetically (i.e., experiences can endow emotions with meaningful associations). Moreover, color involves raw evaluative processes that influence psychological functioning in an automatic way and take place below the individual’s level of consciousness. There are a great many findings to support this theoretical perspective, with the color red being particularly well documented. Studies adopting a range of different procedures have revealed that even the very subtle presence of a red feature in the environment can be detrimental in an achievement context [35]–[38]. The explanation for this negative effect is that red is negatively valenced, and linked to danger, failure (e.g., [39]) and anger (e.g., [40]).

Green is a color that has received less attention, but as it lies directly opposite red in the color spectrum [41], it has often been used in experimental studies as a control or to convey the opposite meaning to red (e.g., [32], [35]). All the findings suggest that green is a positively valenced color, signifying pleasantness, calmness and happiness. For example, red has been shown to enhance memory for negative words, whereas green increases it for positive ones [42]. Other findings also support the positive meaning of green, showing that it promotes creativity [43], or seems to evoke safety [44]. Similarly, it has been suggested that green is associated with growth and fertile natural environments–an association that Akers and collaborators illustrated with the green exercise effect [45]. These authors showed that participants engaged in exercise (i.e., cycling) were in a more positive mood, and felt they were making less effort when they were exposed to a video presenting a green outdoor environment as opposed to a gray- or red-filtered one.

The originality of the present study lay in its focus on positive hue–meaning associations for green, consistent with previous studies, as well as for another color that has so far been neglected in empirical research, even though it has a strong symbolism in Western societies, namely pink. Pink has a strong symbolic association with femininity that is frequently exploited in the arts and marketing [46]. This femininity marker is thought to be related to sweetness, and as suggested in many languages and illustrated by the popular song *La Vie en Rose* (Piaf, 1947), pink also seems to be linked to hope, optimism, happiness and affiliation. Although it is not well documented, there are some findings to back these associations up. For instance, after being exposed to violent and tragic stories, participants tend to be less upset when they fill out a questionnaire on pink paper than when they fill one out on blue or white paper [47]. Along the same lines, pink is seen as referring to desire, happiness and wellbeing [30].

To our knowledge, only two studies have so far examined the influence of colored backgrounds on the perception of facial expressions. Young and colleagues showed that, compared with a green or an achromatic one, a red background facilitates the categorization of angry faces [48]. By contrast, they failed to show that green facilitates the categorization of happy ones. Frühholz and colleagues (2011) examined the impact of color on the recognition of facial expressions of fear, happiness and neutrality, after participants had undergone a learning phase designed to artificially induce a specific association between color and emotion (Experiments 1 and 2) [49]. Importantly for our purpose, the authors stressed that the face–color associations they created were not random, but based on shared emotional properties (i.e., arousal and valence). Their findings revealed a general interference effect in the valenced face categorization task, with increased response times and decreased response accuracy in incongruent trials (i.e., where face and color had not been emotionally associated in the learning phase) compared with congruent ones. They therefore ran a third experiment, in which they switched the face–color combinations around. In other words, the face–color associations created in the learning phase no longer had shared emotional properties. In this condition, results failed to reveal any significant interference effect. Taken together, these three experiments yielded evidence that color, which has low-level perceptual properties [50], can interfere with emotional face processing, and that this interference stems not from those perceptual features but from the color’s emotional charge. As the authors commented, “if the interference effect has been solely elicited by a ‘non-emotional’ violation of an expected face-color pairing, we probably would have found comparable effects in all experiments irrespective of emotional expressions” (p. 22).

The aim of the present study was to investigate the positive meanings of two colors–green in Experiment 1, and pink in Experiment 2–, using the contextual information effect on face perception. Participants performed a forced, two-choice task, in which they had to indicate whether a morphed face shown against a colored background expressed neutrality or a specific emotion. Ambiguous facial expressions are more liable to be influenced by contextual features (i.e., [20], [24], [49]). According to the hue, saturation, lightness (HSL) color system, our two colors of interest differed only on hue. Because we were examining the positive hue–meaning associations that are assumed to be intrinsically present in green and pink, we used faces that expressed two contrasting discrete emotions formalized in several well-established models of emotion (e.g., [51]), namely happiness and sadness. As with the interference effect observed by Frühholz and colleagues, we argued that if green and pink are indeed positively emotionally charged, then compared with a control color, they would facilitate the identification of happiness (i.e., congruent condition) more than sadness (i.e., incongruent condition). Moreover, we chose two achromatic control backgrounds (i.e., white and gray), as they had been used as control colors in previous studies. We also collected subjective ratings for each color from both discrete (e.g., [51]) and dimensional perspectives (e.g., [52]), via five bipolar Osgood scales (*Fear* vs. *Anger*; *Sadness* vs. *Happiness*; *Negative* vs. *Positive (i.e. valence)*; *Calm* vs. *Arousing (i.e. arousal)*; *Unattractiveness* vs. *Attractiveness*). This subjective task was exactly the same for both Experiments, and participants assessed all four colors of interest.

## Experiment 1: Green, White, and Gray

### Method

#### Participants

Thirty-eight women students (mean age = 18.8, *SD* = 1.37) gave their written informed consent – as required by the “Ouest III” Statutory Ethics Committee (CPP) which approved this research - to take part in the study in exchange for course credits. All participants were screened for normal color vision with the short form (i.e., 9 plates) of the Ishihara Color Vision Test [53]. Participants were randomly assigned to one of the two experimental emotion conditions: neutrality–happiness continuum or neutrality–sadness continuum.

#### Material

A PC controlled the experimental events using E-Prime 1.2 software (Psychology Software Tools, Pittsburg, PA). The screen was placed at a distance of approximately 60 cm, and the “D” and “K” keys on a keypad were used for the responses.

Stimuli consisted of faces enclosed in an oval frame so as to exclude the perception of hair. They were displayed in the center of the screen against a color background. The faces were taken from the empirically valid and reliable Pictures of Facial Affect [54]: the same five female faces featuring a neutral affect (0% expression) gradually morphed into a prototypical emotional expression (i.e., 100% expression) of either happiness (neutrality–happiness continuum) or sadness (neutrality–sadness continuum). Extreme expression values were used in the training phases (i.e. 0% and 100%), and faces gradually morphed from 20% to 80% in seven 10% increments were used in the test phases.

Color backgrounds were created according to the three HSL dimensions. We based their characterization of colors on the HSL system, and did not use a spectrophotometer. Green corresponded to *pure* green (i.e., 120° on the color wheel), and lightness and saturation were strictly controlled so that they both corresponded to 100%. For the achromatic backgrounds, gray corresponded to 50% lightness and 0% saturation; and white to 100% and 0% respectively.

Participants rated the emotional aspects of the four colors on five 9-point Osgood scales: *Fear* vs. *Anger*, *Sadness* vs. *Happiness*, *Negative* vs. *Positive* (i.e., valence), *Calm* vs. *Arousing* (i.e., arousal), and *Attractiveness* vs. *Unattractiveness*. These scales were randomly ordered across participants. For the purpose of these ratings, participants were provided with plates featuring the four colors of interest (green, pink, white, gray). The colors were labeled with letters (A–D) and their order was counterbalanced (i.e., a total of 24 boards were created).

#### Procedure

Participants sat at a table facing the computer screen in an isolated room. They began by performing a forced-choice task in which they had to decide as quickly as possible whether the facial expression on the screen was more similar to an *emotion* (i.e., happiness or sadness, according to the experimental condition) or to *neutrality*, pressing the key that corresponded to their response. Key responses were counterbalanced across participants. There were five blocks of trials, each block featuring the facial expressions of one of the five women. Each block comprised a training phase, in which participants performed ten trials featuring extreme emotional expressions displayed against a white background: five neutral (0%) expressions and five with the prototypical emotional face (100% expression). The same woman’s face was used throughout. This phase occurred in the presence of the experimenter, and allowed participants to become familiar with both the task and the woman’s face. Each trial began with a fixation cross (1000 ms), followed by the onset of the stimulus, which disappeared when the participant gave a response. The intertrial interval was 500 ms. Only data from the test phase were used. The test phase took place in the same conditions as the training phase, except that the experimenter left the room and the participant was shown the same woman’s faces four times for each of the three color backgrounds (i.e., green, white and gray) and for each of the seven morphed expressions (i.e., from 20% to 80%). For each block, participants therefore completed 84 test trials (i.e., 4×3×7), leading to a total of 420 test trials. The order of the blocks was counterbalanced across participants, and trials in both phases were administered in a random order.

After this main task, the participants performed the emotional color ratings. The color plate appeared on the PC screen and they had to indicate how far they thought each color expressed the different emotional dimensions, by placing the corresponding letters on the five Osgood scales. In other words, they had to rate each of the four colors on five emotional dimension continuums. Last, they had to name the four colors from left to right. It should be noted that all the participants described the green, white and gray as *green*, *white* and *gray*.

### Results and Discussion

Using logistic mixed models [55], [56], we analyzed the *emotion* responses (i.e., *happiness* in the neutrality–happiness continuum condition, and *sadness* in the neutrality–sadness one) with SAS version 9.4 (GLIMMIX procedure). As analyses run on reaction times failed to reveal either main or interactive effects of color, we only report results corresponding to *emotion* responses. This point is mentioned in the discussion of Experiment 1.

To accommodate the dependence caused by repeated measures, initial models are parameterized with random intercepts and slopes, along with the covariance between the variance components. In the initial model, emotion response curves were fitted by polynomials of morphing percentage, and with emotion and color and the interaction between these two factors as fixed effects, a random intercept for each participant, and random slopes for women’s pictures, color, and morphing percentage (linear, quadratic, and cubic). Emotion could not be entered as a slope as it was a between-participants term. To reduce the multicollinearity of the effects related to the morphing percentages, this variable was centered so that the median value (50°) corresponded to zero. This variable was not (centered-) reduced in order to interpret the odds ratios (ORs). Random slopes are not included in the model when the variable is between-participants (and all the interactions are with a between-participants term). As the participants’ sensitivity cannot vary as a function of a between-subjects factor, they are only submitted to one modality of this type of variable [57]. In addition, the covariance matrix must be specified in mixed models. The covariance matrix used by default is the variance components matrix (its structure is analogous to that of an ANOVA). The inclusion of random effects in this kind of model sometimes involves the nonconvergence of the model. If convergence problems result from the covariance matrix, then the associated variance with at least one random effect is either null or negative, which means that this effect does not significantly contribute to the best model. The GLIMMIX procedure identifies the problematic effect for convergence and it can thus be removed from the model without affecting the quality of the analysis [58]. Moreover, in order to specify the interactions, we ran multiple comparison tests for each significant result using the least-squares means (LSMEANS) option of the MIXED procedure with Bonferroni adjustment, and the error degrees of freedom were row adjusted (ADJDFE = ROW option).

**Figure 1:**
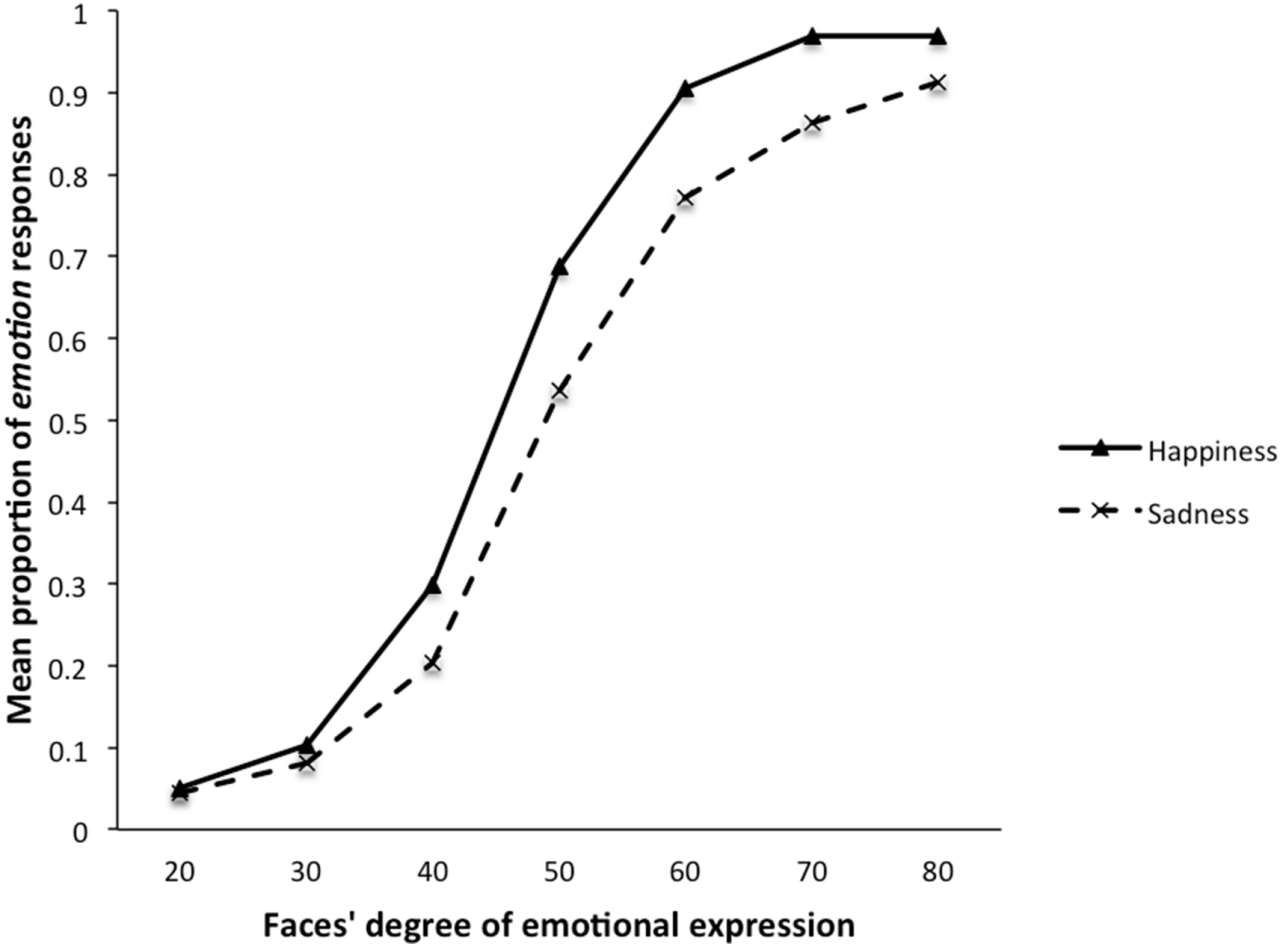
Mean proportion of emotion responses (i.e., happiness or sadness) as a function of the faces’ degree of emotional expression. (Experiment 1).

All other things being equal, the trend in the morphing percentage data was described by a third-order polynomial, or cubic trend (S-curve). The linear, *F*(1, 37) = 24.90, *p*<.001, quadratic, *F*(1, 15654) = −5.66, *p*<.001, and cubic, *F*(1, 15654) = −10.09, *p*<.001, trends significantly described the pattern of the data across the morphing percentages. The OR for a 10% change was 4.751, 95% CI [4.162, 5.423]. Figure 1 shows this typical identification curve for the emotional faces, both curves rising along the continuum of expression. This pattern of data suggested first that participants correctly performed the task, and second that they gave *emotion* responses more frequently in the neutrality–happiness continuum condition than in the neutrality–sadness one.

**Figure 2:**
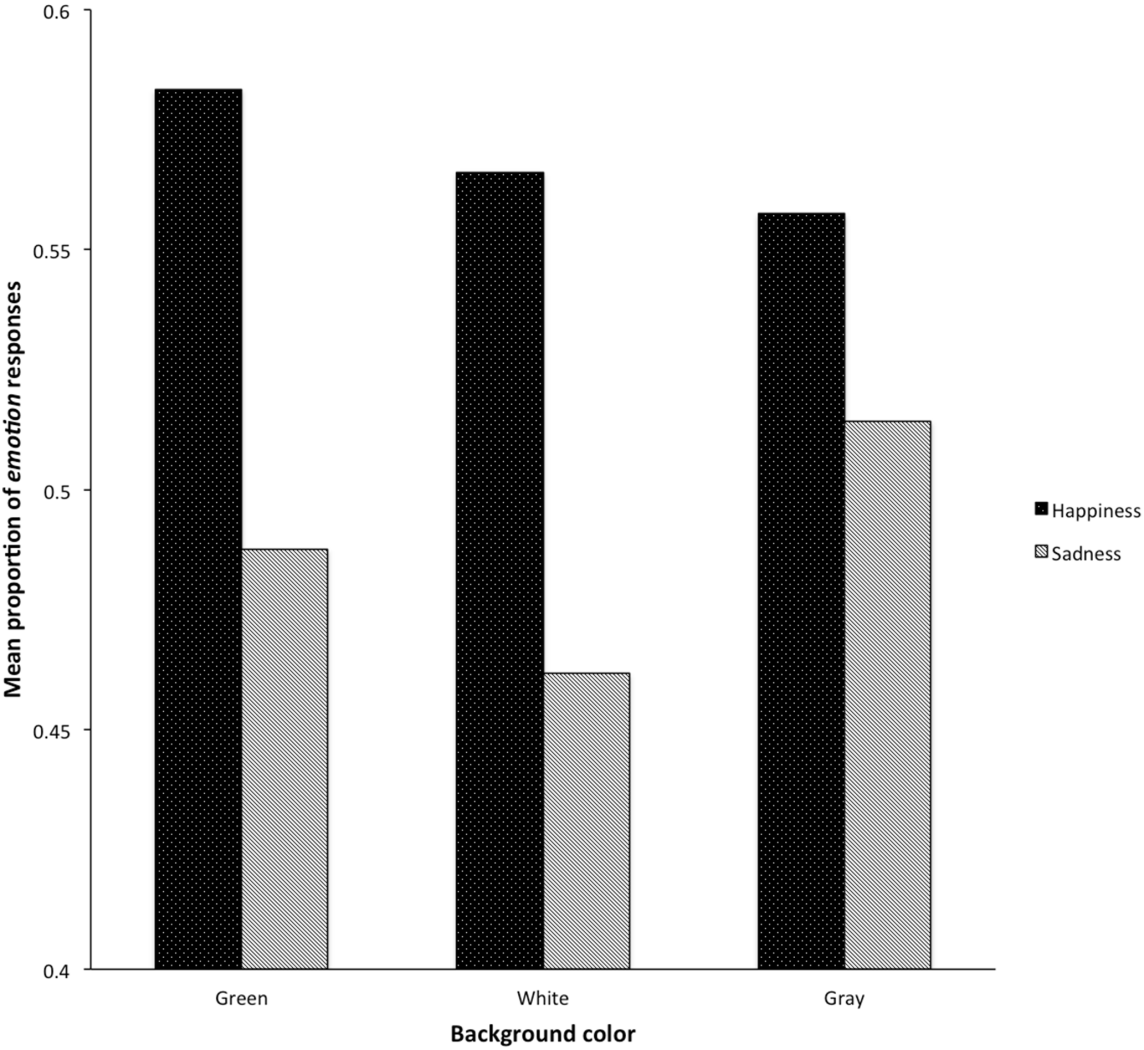
Mean proportion of *emotion* responses according to emotional face and background color (i.e., green, white, or gray).

More importantly for our purpose, the statistical model revealed that emotion, color, and the interaction between the two significantly predicted *emotion* responses, *F*(1, 15654) = 15.59, *p*<.001, *F*(2, 72) = 5.36, *p* = .007, and *F*(2, 15654) = 9.84, *p*<.001 (see Table 1 for the parameters of the logistic mixed model analysis). As Figure 2 illustrated, even if happy faces prompted more emotion responses, this effect was modulated by color. More precisely, multiple comparisons tests revealed that green and white involved significantly more emotion responses for happy faces compared to sad faces (all *ps*<.001), whereas it was not the case for gray (*p* = .60).

**Table 1:** Parameters of the logistic mixed model analysis in Experiment 1.

Effect	Color	Emotion	Estimate	SE	*df*	*t* value	*p*
**Intercept**			0.2820	0.1554	36	1.81	0.0779
**Color**	W		−0.4531	0.09312	72	−4.87	<.0001
**Color**	Gr		−0.2340	0.09301	72	−2.52	0.0141
**Color**	G		0	.	.	.	.
**Emotion**		H	0.4607	0.2202	15654	2.09	0.0364
**Emotion**		S	0	.	.	.	.
Color*Emotion	W	H	0.5417	0.1373	15654	3.95	<.0001
Color*Emotion	W	S	0	.	.	.	.
Color*Emotion	Gr	H	0.5123	0.1373	15654	3.73	0.0002
Color*Emotion	Gr	S	0	.	.	.	.
Color*Emotion	G	H	0	.	.	.	.
Color*Emotion	G	S	0	.	.	.	.
**Degree**			0.1663	0.006679	37	24.90	<.0001
Degree*Degree			−0.00052	0.000091	15654	−5.66	<.0001
Degree*Degree*Degree			−0.00005	5.266E-6	15654	−10.09	<.0001

Emotional faces: H = Happiness, S = Sadness; Background color: Gr = Green, G = Gray, W = White; Degree = morphing percentage.

Based on findings that green has a positive hue–meaning association, we examined whether a green background, as opposed to a control background, would bring about an increase in *emotion* responses to congruent emotional faces (i.e., happy faces) and a decrease in these responses to faces displaying the opposite emotion (i.e., sad faces). The main result bore out our assumption: compared with sad faces, participants gave significantly more *emotion* responses when the morphed expressions of happiness were embedded in a green background rather than a gray one. However, analyses revealed three further results for the face recognition task. First, happy faces were generally better identified than sad ones. This is a well known phenomenon in the area of facial expression research, and is observed with a range of different procedures. As happy faces are characterized by a specific feature (i.e., the smiling mouth), they benefit from a general visual salience that makes them easier to recognize or differentiate from all other expressions (e.g., [59]–[61]). Second, our analyses revealed that background color influenced participants’ responses per se, but not their reaction times. One interpretation would be that we manipulated two kinds of *basic visual object*: emotional faces, which are known to be processed very efficiently, and color, which is a rudimentary physical cue. Consequently, this nonresult may reflect the automaticity of the processing and integration of these two types of cues, doubtless involving a different processing timecourse than studies examining face processing in a more complex context (e.g., emotional scene). Finally, a third unexpected result concerned the impact of white, which had been used as a control background. This result is discussed in the General Discussion.

## Experiment 2: Pink, White, and Gray

### Method

#### Participants

Thirty-eight women students (mean age = 19.2 years; *SD* = 1.35) gave their written informed consent to take part in the study in exchange for course credits. As in Experiment 1, we verified their normal color vision by means of the short form of the Ishihara Color Test [53], and they were randomly assigned to one of the experimental conditions: neutrality–happiness continuum or neutrality–sadness continuum.

#### Material and Procedure

The materials and procedure were similar to those in Experiment 1, except for the background colors: green was replaced by pink, resulting in stimuli where faces were superimposed on pink, white, or gray backgrounds. According to the three HSL dimensions, pink corresponded to a *pure* magenta (300° on the color wheel), and was similar in lightness and saturation to the green in Experiment 1 (i.e., 100%). It should be noted that all participants described the white and gray as *white* and *gray,* and that while 86% of them also described the pink as *pink* or similar (i.e., only 5% described it as magenta or fuchsia), 14% called it purple or deep purple.

### Results and Discussion

Analyses were conducted with SAS version 9.4 (GLIMMIX procedure), and the logistic mixed model for *emotion* responses was exactly the same as in Experiment 1. Moreover, as in Experiment 1, analyses run on reaction times failed to reveal either main or interactive effects of color: we only report results corresponding to *emotion* responses.

**Figure 3:**
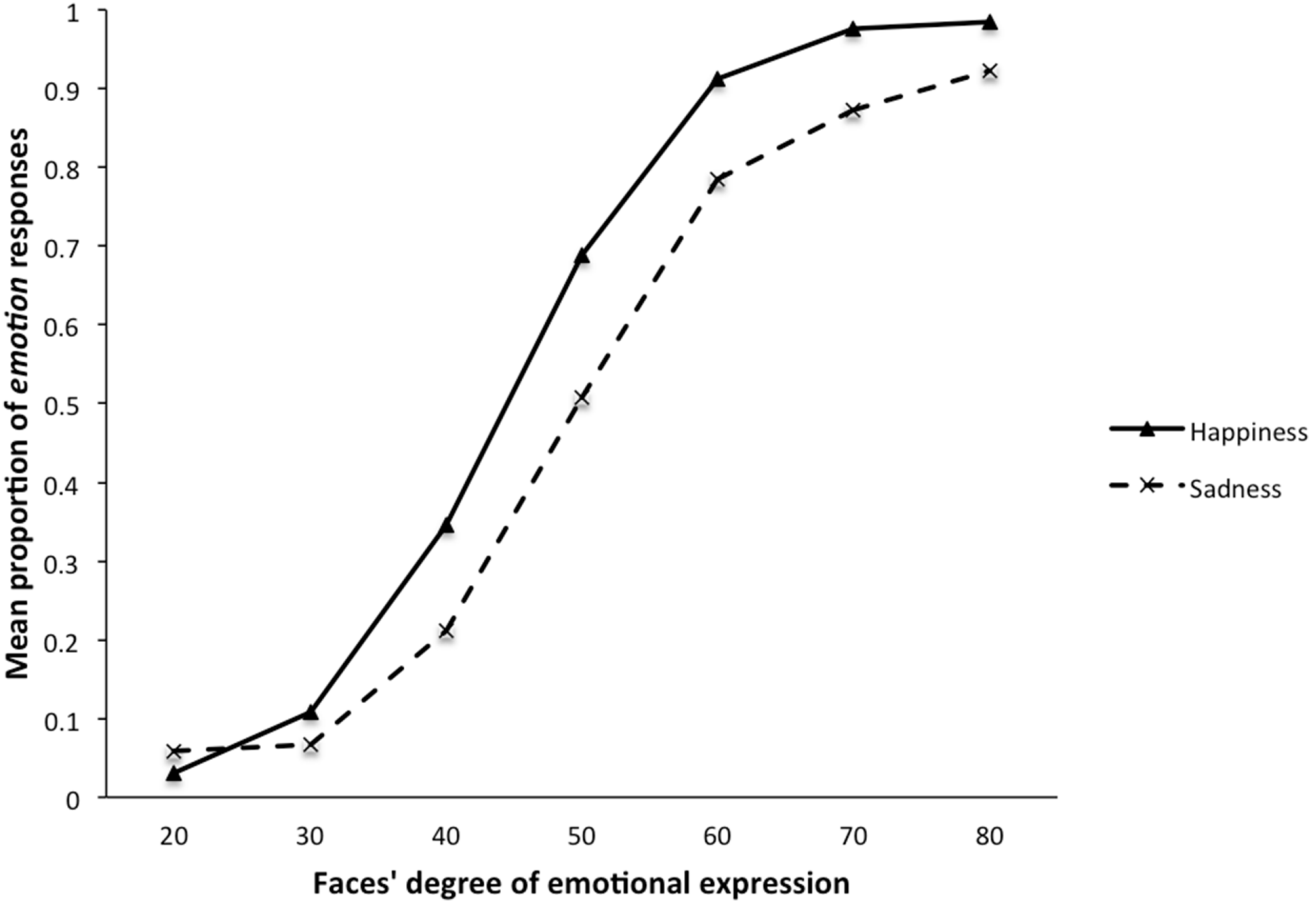
Mean proportion of *emotion* responses (i.e., happiness or sadness) as a function of the faces’ degree of emotional expression. (Experiment 2).

All other things being equal, the trend in the morphing percentage data is described by a third-order polynomial (S-curve). The linear, *F*(1, 37) = 23.65, *p*<.001, quadratic, *F*(1, 15654) = −4.07, *p*<.001, and cubic, *F*(1, 15654) = −8.93, *p*<.001, trends significantly described the pattern of the data across the percentages (Figure 3). The OR for a 10% change was 5.396, 95% CI [4.646, 6.266].

**Figure 4:**
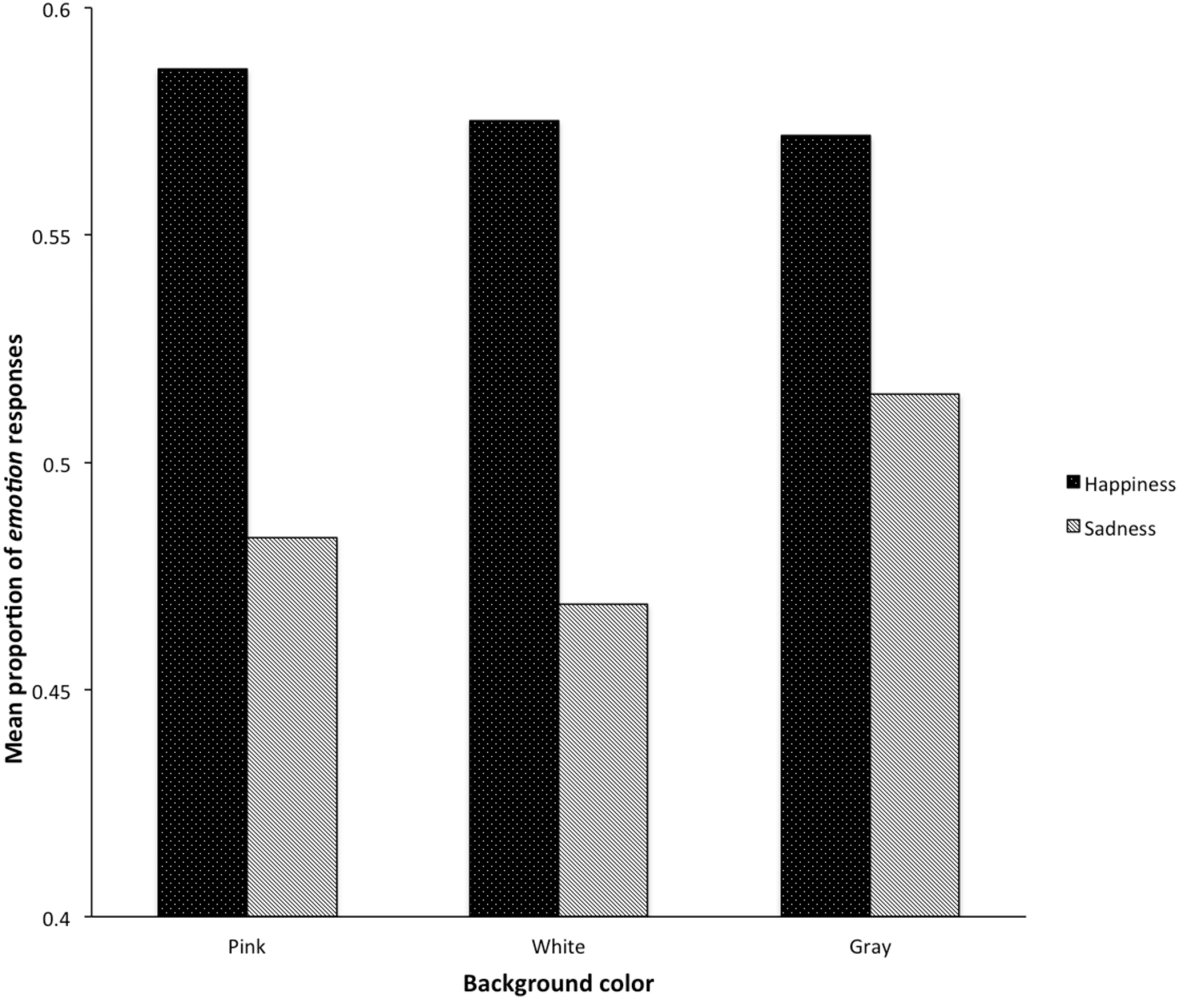
Mean proportion of *emotion* responses according to emotional face and background color (i.e., pink, white, or gray).

Emotion, Color and the Emotion×Color interaction predicted *emotion* responses, *F*(1, 15654) = 10.95, *p* = .001, *F*(2, 72) = 3.61, *p* = .03, and *F*(2, 15654) = 6.98, *p* = .001 (see Table 2 for the parameters of the logistic mixed model analysis). As in Experiment 1, even happy faces prompted more emotion responses, this effect depended on color background: pink and white therefore modulated the ability to discriminate emotional faces compared with gray (Figure 4). *Emotion* responses to happy faces compared with sad faces were significantly different for pink (*p* = .003) and white (*p* = .003), but not for gray (*p* = .45).

**Table 2:** Parameters of the logistic mixed model analysis in Experiment 2.

Effect	color	emotion	Estimate	SE	DF	t Value	p
**Intercept**			0.2912	0.2157	36	1.35	0.1853
**color**	W		−0.4175	0.09571	72	−4.36	<.0001
**color**	P		−0.2878	0.09562	72	−3.01	0.0036
**color**	G		0	.	.	.	.
**emotion**		H	0.6648	0.3063	15654	2.17	0.0300
**emotion**		S	0	.	.	.	.
color*emotion	W	H	0.4603	0.1442	15654	3.19	0.0014
color*emotion	W	S	0	.	.	.	.
color*emotion	P	H	0.4728	0.1442	15654	3.28	0.0010
color*emotion	P	S	0	.	.	.	.
color*emotion	G	H	0	.	.	.	.
color*emotion	G	S	0	.	.	.	.
**degree**			0.1776	0.007512	37	23.65	<.0001
degree*degree			−0.00041	0.000100	15654	−4.07	<.0001
degree*degree*degree			−0.00005	5.595E-6	15654	−8.93	<.0001

Emotional faces: H = Happiness, S = Sadness; Background colors: P = Pink, G = Gray, W = White; Degree = morphing percentage.

In sum, data from Experiment 2 were very close to the pattern of results in Experiment 1. Confirming our assumption, when pink was used as a background for face processing, it facilitated the identification of happy facial expressions compared with sad ones. This result corresponded to our hypothesis that information processing benefits from a congruent context, and confirmed the positive hue–meaning association for pink.

## Experiments 1 & 2: Subjective Color Ranking

**Figure 5:**
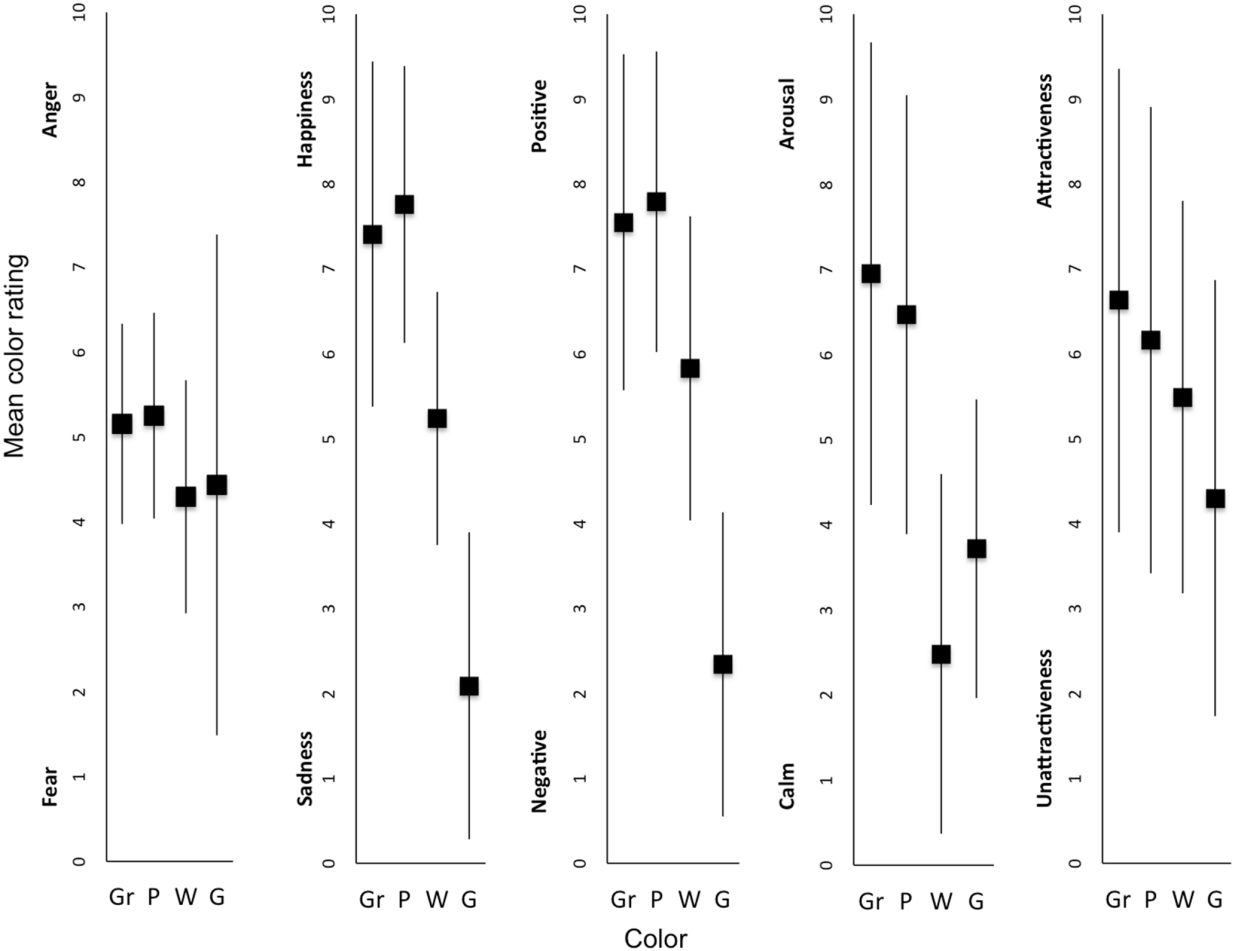
Color ratings (from left to right: *Fear* vs. *Anger*, *Sadness* vs. *Happiness*, *Negative* vs. *Positive*, *Calm* vs. *Arousing*, *Unattractiveness* vs. *Attractiveness*). Gr = Green, P = Pink, W = White, G = Gray.

After performing the face recognition task, the participants rated the colors on the five bipolar Osgood scales. This resulted in a classification of colors on each continuum. Figure 5 illustrates these classifications, which were subjected to nonparametric tests, using Friedman’s analysis of variance and the Wilcoxon signed-rank test for pairwise comparisons.

Analysis revealed a significant effect of color on the fear–anger continuum classifications, *Chi^2^*(3) = 20.04, *p*<.001. White (*M* = 4.29, *SD* = 1.373) and gray (*M* = 4.43, *SD* = 2.95) were rated as being closer to fear than pink (*M* = 5.25, *SD* = 1.209) or green (*M* = 5.15, *SD* = 1.182) (all *p*s<.05). The differences between white and gray, and between pink and green, were not significant (all *p*s>.554).

There was a significant effect of color on the sadness–happiness continuum classifications, *Chi^2^*(3 = 147.65 *p*<.001. Pairwise comparisons showed that gray (*M* = 2.09, *SD* = 1.81) was judged to be closest to sadness, and differed significantly from white (*M* = 5.24, *SD* = 1.487) which, in turn, differed from both pink (*M* = 7.76, *SD* = 1.626) and green (*M* = 7.41, *SD* = 2.034) (all *p*s<.001). Pink and green therefore appeared to be closest to happiness, and did not differ from each other (*p* = .054).

There was a significant effect of color on the valence continuum classifications, *Chi^2^*(3) = 141.43, *p*<.001. Gray was classified as the most negative color (*M* = 2.34, *SD* = 1.792), followed by white (*M* = 5.83, *SD* = 1.792), which differed from both pink (*M* = 7.79, *SD* = 1.769) and green (*M* = 7.55, *SD* = 1.983) (all *p*s<.001). Pink and green did not differ significantly from each (*p* = .320), both being judged as the most positive. As expected, the valence and sadness–happiness continuums yielded similar color evaluations.

Concerning the arousal dimension, results revealed a significant effect of color on participants’ judgments, *Chi^2^*(3) = 88.347, *p*<.001. White was judged to be the least arousing color (*M* = 2.48, *SD* = 2.114), followed by gray (*M* = 3.72, *SD* = 1.752), then pink (*M* = 6.47, *SD* = 2.58) and green (*M* = 6.95, *SD* = 2.716) (all *p*s<.001). Pink and green did not differ significantly from each other (*p* = .103), and were therefore judged to be the most arousing colors.

Finally, color had a significant effect on the attractiveness dimension, *Chi^2^*(3) = 31.76, *p*<.001. Pairwise comparisons revealed that gray (*M* = 4.30, *SD* = 2.567) was judged to be the least attractive color (all *p*s<.001). Moreover, white (*M* = 5.49, *SD* = 2.312) was judged to be significantly less attractive than green (*M* = 6.63, *SD* = 2.732), whereas pink (*M* = 6.16, *SD* = 2.743) did not differ significantly from either white (*p* = .217) or green (*p* = .507).

## General Discussion

Based on research showing that emotional contextual information can influence how humans process emotional faces [16], [17], we set out to examine the impact of color as one such source of information. There has been a recent surge of interest in color among psychologists, reflecting the fact that color is as an omnipresent feature of our environment and therefore presumably plays a role in information processing. Up to now, studies have mainly focused on the color red, showing that red can heighten negative emotions, such as failure and danger (e.g., [37]–[39], [62]). To a lesser extent–and often contrasting it with red–studies have revealed that green conveys a positive meaning (e.g., [35], [42], [43], [62]). The originality of our study was to focus on two colors that we assumed to be positively charged, namely green and a color that has received even less attention from researchers: pink.

To this end, we administered an emotional facial expression recognition task in which the faces were displayed against a color background. Two experiments allowed us to investigate the two colors separately, but using similar procedures. These involved the use of morphed emotional faces, which allowed us to use stimuli that varied in the extent to which they elicited the intended emotion. In each experiment, we used two conditions: a neutrality–happiness continuum and a neutrality–sadness continuum, the former representing an emotion that was congruent with green and pink, the latter one that was incongruent. Analyses yielded very close patterns of results for the two colors under investigation. Compared with the gray control background, the green and pink backgrounds prompted better recognition of happy faces than of sad ones. Our experiments therefore yielded some interesting data for two positively charged colors that have been relatively undocumented, and our findings were consistent with previous research on green and with the handful of studies of pink [30], [47]. Because both green and pink convey positive information, they can be assumed to play a facilitating role in the processing of emotionally congruent facial expressions (i.e., faces expressing happiness), and conversely an interfering role in the processing of incongruent expressions (i.e., faces expressing sadness). Our findings also confirm that a basic cue environment, such as color, can influence the processing of emotional faces [49] and, more importantly, show that this impact may be due to intrinsic color–meaning associations. Moreover, compared with Young and colleagues’ investigation of red [48], our experimental design featured a more constrained color–face processing environment, as the two sources of information were presented concomitantly (i.e., no color priming). Finally, as far as meaning is concerned, the results of the emotional face recognition task (i.e., an implicit color–meaning measure) were sustained by the color ratings (i.e., an explicit color–meaning measure), as participants judged both pink and green to evoke positive emotions in general, and happiness in particular.

One unexpected result concerned the effect of white, an achromatic color we had initially chosen as a control, on the strength of various recent studies that had used it as a control condition because of its achromatic nature (e.g., [35]). Contrary to our expectations, findings for both experiments showed that white influenced the face processing in a similar manner to green and pink, thus suggesting that it is associated with a positive meaning. The color white is not well documented in the psychology literature, in terms of its potential emotional meaning, reflecting the fact that it has tended to be regarded simply as a control condition up to now. However, two strands of research can inform and make sense of our results. First, in design and ergonomics research, it is possible to find results showing that the impact of white on wellbeing and performance differs according to the context (e.g., [63], [64]). White has usually been considered in the light of its achromatic nature in these kinds of studies, the idea being that it can either improve efficiency because it is less distracting than chromatic colors, or result in poorer performances because it is less arousing than chromatic colors. The color ratings given by our participants support the latter interpretation, for when we compared the results of the implicit emotional face recognition task and the explicit color ratings, we found that white had a different pattern of results from green and pink: participants explicitly judged it to be the most relaxing color, conveying tranquility, but devoid of valence and emotion. Second, and more important for our purpose, there is considerable evidence to show that one particular dimension of color, namely lightness, which can be regarded as one of the defining characteristics of achromatism, has an intrinsic emotional meaning [65]. White, of course, has a high level of lightness, and this physical property has been shown to have a positive meaning: dark objects seem bad, whereas bright objects seem good (e.g., [66], [67]). Meier and colleagues, for instance, showed that the categorization of positive (vs. negative) words is enhanced when they are printed against a white (vs. black) background [68], and in turn that a gray square is judged to be brighter when its presentation is preceded by a positive word rather than a negative one [69]. In a similar vein, a recent study showed that a commonly used set of emotional pictures, the International Affective Picture System (IAPS) [70] can be a good illustration of this brightness bias [71].

In brief, as well as the influence of hue per se, other color dimensions may have an impact on psychological functioning. This heuristic point raises questions about what researchers can use as a *control color*, and the power of color relative to another. From the observed pattern of results (i.e., white yielding similar results to green and pink), one alternative interpretation is that lightness plays a role in how color backgrounds evoke positive emotion [65]. Crucially, it could be argued that by adopting a procedure based on congruency between two sources of emotional information–emotional faces being the known source, and color backgrounds the tested source–, we have gone some way toward disentangling this issue. In other words, our results converge strongly with previous experimental findings, and further confirm the effect of colored backgrounds on face processing by revealing two different patterns of enhancement: happy face processing enhanced by green, pink and white more than sad face processing. However, it may conceivably have been the combination of hue and lightness that resulted in a positive meaning. Further work along these lines is needed to determine the respective influence of these two dimensions: is *La vie en rose* positive because of the hue or because the hue is also bright? In contrast to white, our results suggest that a gray background does indeed constitute a control condition, in that gray has no specific emotional meaning: we found no difference in its impact on one emotional expression relative to another. Nonetheless, one alternative interpretation is that gray has a negative hue–meaning association, in which case it would have influenced our results, producing a sort of contrast effect. Given the literature about the impact of lightness discussed above, this idea seems plausible, as gray was somewhat darker than the other colors we used. Moreover, it converges with the results of the subjective color ratings, which showed that gray was viewed as conveying negative emotion and sadness, in contrast to the positivity and happiness conveyed by both pink and green. In turn, these ratings are consistent with the results of other studies featuring self-report questionnaires (e.g., [72]). However, although we cannot reject this interpretation, it does not fit the present statistical pattern of results. If gray had a negative meaning, the interaction between color and emotion would be an antagonistic one, not a multiplicative one. Our results showed that pink, green and white backgrounds systematically involved more emotion responses than the gray background for happy faces compared with sad ones, but we did not observe any difference in the impact of the gray background for one emotional face relative to another. In other words, gray backgrounds appeared not to facilitate the recognition of expressions of either happiness or sadness. Moreover, the idea that gray is neutral converges with the study by Bonnardel and collaborators [31], which showed that, compared with a panel of test colors, gray allowed participants to carry out more efficient web information searches, regardless of the emotional nature of that information.

To conclude, our findings show that green, pink and white backgrounds implicitly modulated how participants processed emotional faces, facilitating happy face processing and hindering sad face processing. Our findings thus linked these colors to expressions of happiness but not of sadness, the theoretically opposite emotion (e.g., [51]). However, future research will need to determine whether we should talk about a color–emotion association from a discrete or a dimensional perspective. In other words, are some colors related to happiness in particular or to positive emotions in general? Emotion scientists adopt two major approaches to characterizing emotions: dimensional and discrete. According to the dimensional approach, emotion can be generally–though not exclusively–characterized by two restrictive dimensions: valence and arousal (e.g., [52], [73]). The discrete approach, on the other hand, assumes that a dimensional approach can only reflect the basic nature of affect, and emotions are therefore conceptualized not as a function of valence and arousal, but in relation to their specific constituent features and functions (e.g., fear and sadness) [5], [9]. According to this account, if color–emotion associations do exist, further research is needed to understand whether colors are associated with valence or with discrete emotions. For instance, some studies investigating the color red in relation to two similarly (negative) valenced emotions, namely anger and sadness [40] or anger and fear [48], showed that red is only related to anger.

Finally, the color-in-context theory developed by Elliot and colleagues [35] suggests that the same color can take on different meanings in different contexts. For instance, red is deleterious in an achievement context [38], but increases the perceived attractiveness of a member of the opposite sex (e.g., [74], [75]). It might well be worthwhile exploring the sex of participants and face gender factors, and their interaction with color–meaning associations in future research, as our study, featuring solely female faces and solely female participants, was not designed to examine this issue. Linked to this notion, color–meaning associations could have two origins: evolutionary or cultural. In the case of the two colors we studied, which of these interpretations is the most relevant? It is very difficult to answer this question, as phylogenetic and ontogenetic mechanisms are intrinsically linked. Nonetheless, we can argue for an a priori difference between green and pink. Green (like red) can refer both to the natural features of our environment (e.g., vegetation and fertile growth) and to culturally embedded environmental cues (e.g., green light for motorists). In contrast, pink seems to be linked more to cultural learning, and is thus potentially influenced more by context. Although they do not distinguish between these two developmental perspectives, intercultural studies seem to emphasize the importance of life experience (i.e., implicit learning) for color meanings. For instance, a recent study showed that Mainland Chinese and Hong Kong citizens (i.e., two populations belonging to the same society, but with different cultures) were influenced by green and red in contrasting ways in economic judgments, because these two colors are associated with opposite meanings in each culture (i.e., growth vs. decline) [76]. Moreover, the question of the origin of color–meaning associations raises the question of which processes are involved in this crossmodal correspondence [65], [77], and how humans manage its cognitive representations [68], [69].

In relation to the idea that people use unbounded sources of information, and that information processing gains from congruent sources, our overall findings suggest that green and pink are associated with positive meanings, both explicitly and implicitly, thus impacting on how we perceive our environment. As far as the psychology of color meaning is concerned, the results of the present study, based on an original experimental design, therefore support the hue–meaning association hypothesis. However, the spectrum of color–meaning associations, along with the precise nature of the mechanisms that underpin them, remains unclear and requires further investigation. Extending this research to the exploration of hitherto neglected colors and using new methodologies would doubtless enhance current understanding in this exciting area of research.

## Supporting Information

Table S1 **Experiment 1.** Mean proportion of emotion responses for each emotional expression and each color, per participant (140 trials for each experimental condition).
(PDF)

Table S2 **Experiment 2.** Mean proportion of emotion responses for each emotional expression and each color, per participant (140 trials for each experimental condition).
(PDF)
