# Supplementary material for: Seeing Life through Positive-Tinted Glasses: Color–Meaning Associations
Source: PLoS One. 2014 Aug 6;9(8):e104291. doi: 10.1371/journal.pone.0104291 (PMC4123920; doi:10.1371/journal.pone.0104291)
Supplement: Table S1 — Experiment 1. Mean proportion of emotion responses for each emotional expression and each color, per participant (140 trials for each experimental condition). (PDF) [file pone.0104291.s001.pdf]

	Happiness			Sadness		
Participant	Green	White	Gray	Green	White	Gray
1	0.65	0.7	0.67857143			
2	0.5	0.52857143	0.52142857			
3	0.62857143	0.63571429	0.60714286			
4				0.52142857	0.52142857	0.50714286
5				0.48571429	0.50714286	0.53571429
6				0.42142857	0.38571429	0.47857143
7				0.49285714	0.45	0.42857143
8	0.75714286	0.71428571	0.62857143			
9	0.48571429	0.43571429	0.46428571			
10	0.56428571	0.50714286	0.52142857			
11	0.57857143	0.53571429	0.54285714			
12				0.55	0.53571429	0.55714286
13				0.42857143	0.36428571	0.4
14				0.50714286	0.52142857	0.55
15				0.29285714	0.37857143	0.39285714
16	0.52857143	0.5	0.52142857			
17	0.57857143	0.57142857	0.58571429			
18	0.62142857	0.6	0.57857143			
19	0.55714286	0.45714286	0.45			
20	0.60714286	0.57142857	0.58571429			
21				0.54285714	0.4	0.52857143
22				0.44285714	0.33571429	0.47857143
23				0.45714286	0.42857143	0.50714286
24				0.46428571	0.39285714	0.43571429
25				0.46428571	0.50714286	0.53571429
26	0.49285714	0.52142857	0.51428571			
27	0.62857143	0.55714286	0.57142857			
28	0.62142857	0.60714286	0.57142857			
29				0.57142857	0.57857143	0.57142857
30				0.54285714	0.57142857	0.55
31				0.52142857	0.45714286	0.6
32	0.54285714	0.55714286	0.53571429			
33	0.55	0.6	0.54285714			
34	0.65714286	0.65714286	0.67142857			
35	0.53571429	0.5	0.5			
36				0.48571429	0.49285714	0.52142857
37				0.51428571	0.48571429	0.58571429
38				0.55714286	0.45714286	0.60714286
