# Supplementary material for: Seeing Life through Positive-Tinted Glasses: Color–Meaning Associations
Source: PLoS One. 2014 Aug 6;9(8):e104291. doi: 10.1371/journal.pone.0104291 (PMC4123920; doi:10.1371/journal.pone.0104291)
Supplement: Table S2 — Experiment 2. Mean proportion of emotion responses for each emotional expression and each color, per participant (140 trials for each experimental condition). (PDF) [file pone.0104291.s002.pdf]

	Happiness				Sadness		
Participant	Pink	White	Gray		Pink	White	Gray
1					0.51428571	0.46428571	0.50714286
2					0.42142857	0.43571429	0.46428571
3					0.54285714	0.56428571	0.57142857
4					0.45	0.38571429	0.49285714
5					0.43571429	0.43571429	0.45
6	0.49285714	0.5	0.45				
7	0.67142857	0.62142857	0.65				
8	0.54285714	0.59285714	0.57857143				
9	0.55	0.54285714	0.55				
10					0.49285714	0.47142857	0.51428571
11					0.67857143	0.67142857	0.71428571
12	0.69285714	0.67857143	0.66428571				
13	0.55714286	0.51428571	0.52142857				
14	0.54285714	0.52857143	0.57857143				
15	0.66428571	0.65714286	0.62142857				
16					0.39285714	0.36428571	0.38571429
17					0.34285714	0.42142857	0.41428571
18					0.54285714	0.46428571	0.54285714
19					0.50714286	0.55	0.57142857
20					0.52142857	0.48571429	0.52142857
21	0.64285714	0.64285714	0.65				
22	0.53571429	0.55	0.54285714				
23	0.55714286	0.56428571	0.52857143				
24					0.52857143	0.51428571	0.56428571
25					0.40714286	0.38571429	0.45714286
26					0.6	0.48571429	0.55714286
27	0.55	0.54285714	0.53571429				
28	0.52857143	0.52142857	0.57857143				
29	0.55	0.49285714	0.51428571				
30	0.60714286	0.55	0.55				
31					0.42857143	0.35	0.43571429
32					0.54285714	0.58571429	0.5
33					0.39285714	0.40714286	0.6
34	0.52142857	0.54285714	0.50714286				
35	0.65714286	0.66428571	0.62142857				
36	0.52142857	0.47142857	0.45714286				
37	0.75714286	0.75	0.76428571				
38					0.44285714	0.46428571	0.52142857
